# Supplementary material for: A fault-intrusion-tolerant system and deadline-aware algorithm for scheduling scientific workflow in the cloud
Source: PeerJ Comput Sci. 2021 Nov 2;7:e747. doi: 10.7717/peerj-cs.747 (PMC8576554; doi:10.7717/peerj-cs.747)
Supplement: Supplemental Information 6 [file peerj-cs-07-747-s006.zip › Myproject1.1/docs/org/workflowsim/CondorVM.html]

CondorVM


JavaScript is disabled on your browser.


- Overview
- Package
- Class
- Use
- Tree
- Deprecated
- Index
- Help

- Prev Class
- Next Class

- Frames
- No Frames

- All Classes

- Summary:
- Nested |
- Field |
- Constr |
- Method

- Detail:
- Field |
- Constr |
- Method


org.workflowsim

## Class CondorVM

- java.lang.Object
- - org.cloudbus.cloudsim.Vm
  - - org.workflowsim.CondorVM

- ---

    

  ```
  public class CondorVM
  extends Vm
  ```

  Condor Vm extends a VM: the difference is it has a locl storage system and it
  has a state to indicate whether it is busy or not

  Since:
  :   WorkflowSim Toolkit 1.0

- - ### Constructor Summary

    Constructors

    Constructor and Description

    `CondorVM(int id,
    int userId,
    double mips,
    int numberOfPes,
    int ram,
    long bw,
    long size,
    java.lang.String vmm,
    CloudletScheduler cloudletScheduler)`

    Creates a new CondorVM object.
  - ### Method Summary

    Methods

    | Modifier and Type | Method and Description |
    | --- | --- |
    | `void` | `addLocalFile(File file)` Adds a file to the local file system |
    | `int` | `getState()` Gets the state of the task |
    | `boolean` | `hasLocalFile(File file)` Tells whether a file is in the local file system |
    | `void` | `removeLocalFile(File file)` Removes a file from the local file system |
    | `void` | `setState(int tag)` Sets the state of the task |

    - ### Methods inherited from class org.cloudbus.cloudsim.Vm

      `addStateHistoryEntry, getBw, getCloudletScheduler, getCurrentAllocatedBw, getCurrentAllocatedMips, getCurrentAllocatedRam, getCurrentAllocatedSize, getCurrentRequestedBw, getCurrentRequestedMaxMips, getCurrentRequestedMips, getCurrentRequestedRam, getCurrentRequestedTotalMips, getHost, getId, getMips, getNumberOfPes, getRam, getSize, getStateHistory, getTotalUtilizationOfCpu, getTotalUtilizationOfCpuMips, getUid, getUid, getUserId, getVmm, isBeingInstantiated, isInMigration, setBeingInstantiated, setBw, setCloudletScheduler, setCurrentAllocatedBw, setCurrentAllocatedMips, setCurrentAllocatedRam, setCurrentAllocatedSize, setHost, setId, setInMigration, setMips, setNumberOfPes, setRam, setSize, setUid, setUserId, setVmm, updateVmProcessing`
    - ### Methods inherited from class java.lang.Object

      `clone, equals, finalize, getClass, hashCode, notify, notifyAll, toString, wait, wait, wait`

- - ### Constructor Detail


    - #### CondorVM

      ```
      public CondorVM(int id,
              int userId,
              double mips,
              int numberOfPes,
              int ram,
              long bw,
              long size,
              java.lang.String vmm,
              CloudletScheduler cloudletScheduler)
      ```

      Creates a new CondorVM object.

      Parameters:
      :   `id` - unique ID of the VM
      :   `userId` - ID of the VM's owner
      :   `mips` - the mips
      :   `numberOfPes` - amount of CPUs
      :   `ram` - amount of ram
      :   `bw` - amount of bandwidth
      :   `size` - amount of storage
      :   `vmm` - virtual machine monitor
      :   `cloudletScheduler` - cloudletScheduler policy for cloudlets
  - ### Method Detail


    - #### setState

      ```
      public final void setState(int tag)
      ```

      Sets the state of the task

      Parameters:
      :   `type` - the type


    - #### getState

      ```
      public final int getState()
      ```

      Gets the state of the task

      Returns:
      :   the state of the task


    - #### addLocalFile

      ```
      public void addLocalFile(File file)
      ```

      Adds a file to the local file system

      Parameters:
      :   `file` - to file to be added to the local


    - #### removeLocalFile

      ```
      public void removeLocalFile(File file)
      ```

      Removes a file from the local file system

      Parameters:
      :   `file` - to file to be removed to the local


    - #### hasLocalFile

      ```
      public boolean hasLocalFile(File file)
      ```

      Tells whether a file is in the local file system

      Returns:
      :   whether the file exists in the local file system


- Overview
- Package
- Class
- Use
- Tree
- Deprecated
- Index
- Help

- Prev Class
- Next Class

- Frames
- No Frames

- All Classes

- Summary:
- Nested |
- Field |
- Constr |
- Method

- Detail:
- Field |
- Constr |
- Method
